# The Influence of Spinning Process on the Properties and Structure of PBS Fibers

**DOI:** 10.3390/polym17091138

**Published:** 2025-04-22

**Authors:** Hao Liu, Hui Li, Zexu Hu

**Affiliations:** State Key Laboratory of Advanced Fiber Materials, Center for Advanced Low-dimension Materials, College of Materials Science and Engineering, Donghua University, Shanghai 201620, China; liuhao6917@163.com

**Keywords:** PBS pre-oriented yarns, spinning processes, mechanical properties, spherulites, lamellae

## Abstract

As a bio-based polymer, polybutylene succinate (PBS) has extensive applications in plastic products and film manufacturing. However, its low melt strength results in poor spinnability, and during the forming process, it tends to form large-sized spherulites and exhibit filament adhesion phenomena. These limitations have hindered its development in the field of fiber spinning. To enhance fiber strength, this work systematically investigated the effects of spinning temperature and spinning speed on the properties and structure of PBS pre-oriented yarns (PBS-POY). The results indicated that appropriately lowering the spinning temperature and increasing the spinning speed could improve the mechanical properties of the fibers. When the spinning temperature was 195 °C and the spinning speed reached 2500 m/min, the tensile strength of pre-oriented yarns achieved 2.09 cN/dtex. Furthermore, the evolution of properties and structures of pre-oriented yarns under maximum drawing conditions across different spinning speed systems was examined. By synchronously analyzing the correlations among mechanical properties, thermal behavior and condensed state structures, the structural performance regulation mechanism under the synergistic effect of spinning–drawing processes was revealed. The results demonstrated that fibers produced at higher spinning speeds contained more numerous and smaller spherulites. After maximum drawing, these smaller spherulites split into lamellae with higher uniformity, resulting in final fibers with smaller crystal sizes, higher crystallinity and improved orientation. As the spinning speed increased, the average crystal size of the final fibers decreased; the long period of the final fibers extended from 8.55 nm to 9.99 nm, and the mechanical strength improved to 2.72 cN/dtex.

## 1. Introduction

In recent decades, petroleum-based plastics have experienced exponential growth across multiple sectors [1,2], including agriculture [3,4,5], industrial manufacturing [6,7,8], construction [9,10,11] and healthcare [12,13,14]. While these materials have significantly contributed to technological advancement, their pervasive use has precipitated two critical global challenges: resource depletion [15] and environmental contamination [16]. This dual crisis has intensified the search for sustainable alternatives, propelling biodegradable materials to the forefront of materials science research [17]. Among emerging biodegradable polymers, polybutylene succinate (PBS) has garnered considerable attention due to its exceptional material properties [18,19,20]. Characterized by high thermal stability [21], broad processing temperature windows [22], remarkable hydrolysis resistance [23] and balanced mechanical performance coupled with inherent biodegradability [24], PBS demonstrates promising potential for applications ranging from food packaging to agricultural films [25]. However, current industrial adoption primarily focuses on molded plastic products [26,27] and thin-film manufacturing [28,29], while its development in the field of melt spinning remains in its early stages. This is due to PBS’s linear molecular chain structure, which lacks long-chain branches or cross-linked networks, resulting in low melt strength and poor spinnability in the molten state. Additionally, PBS exhibits slow nucleation rates and delayed fiber solidification and crystallization rates during processing, leading to the formation of large-sized spherulites and filament sticking during molding. The presence of such large spherulite structures restricts the mechanical properties of the fibers. These factors collectively hinder the advancement of PBS in melt spinning applications [30].

Current research progress in PBS spinning technology remains constrained, with extant literature predominantly concentrating on PBS composite fiber development and performance characterization. In a pivotal study by Wattana et al. [31], innovative hollow segmented pie-shaped PLA/PBS composite fibers were engineered through advanced melt-spinning techniques. Comparative analysis with conventional PLA/PP composites revealed superior phase compatibility in the PLA/PBS system, achieving stable blending ratios up to 50/50 while maintaining morphological stability. This breakthrough establishes a viable pathway for optimizing the tensile strength-elongation balance in biopolymer fiber systems through strategic component formulation. Sao et al. [32] fabricated MP extract-coated PLA/PBS hybrid fibers for functional non-woven textiles. Experimental findings indicated a 23% enhancement in tensile strength following MP incorporation, attributed to effective interfacial stress transfer mediated by the bioactive coating. Significantly, the modified fibers preserved over 90% of their biodegradation efficiency while exhibiting >99% antibacterial efficacy against Staphylococcus aureus, meeting critical requirements for biomedical material applications. This dual functionality paradigm successfully reconciles mechanical reinforcement with biological activity without compromising environmental sustainability.

Additionally, there have been a few reports on pure PBS fiber properties. Morgan [33] investigated PBS monofilaments for fishing gear applications, studying the effects of draw ratio and drawing temperature on fiber performance. The results indicated that PBS monofilaments achieved optimal mechanical properties under a draw ratio of 4.5 and drawing temperature of 80 °C. Chen Yong et al. [34] investigated the relationship between macromolecular entanglement and branched structures on the crystallization and rheological properties of PBS, and prepared PBS multi-filaments. Both strategies strongly influenced the performance of PBS by affecting its crystallinity, crystallization rate, spherulite growth and morphology. The results showed that the presence of a branched structure could significantly enhance the performance of PBS fibers, with the strength of PBS-POY fibers reaching up to 1.62 cN/dtex. Nevertheless, existing research has not resolved PBS’s tendency to generate large spherulites. Hence, it is critical to develop approaches to accelerate the crystallization kinetics of PBS melts, thus minimizing spherulite dimensions.

In fact, existing studies have reported that high-speed spinning methods can regulate polymer crystal structures and properties by enhancing crystallization rates through stress-induced crystallization mechanisms [35,36,37]. Kim [38] investigated structural evolution in high-speed melt-spun polyethylene terephthalate (PET) fibers, revealing that the high spinning tension generated during high-speed spinning significantly accelerates fiber crystallization rates. Furthermore, Song [39] explored the effects of different spinning speeds on the properties of PVA/PA6 sea–island pre-oriented yarns. The results demonstrated that elevated spinning velocities enhanced orientation-induced crystallization behavior, leading to reduced crystalline grain size and markedly improved tensile strength in the fibers. However, current research on the influence of melt spinning production processes on the structure and properties of PBS fibers remains limited, particularly regarding the effects of spinning speed on the post-drawn fiber performance and structural evolution. In the field of melt spinning, the performance of pre-oriented yarns often fell short of application standards, while high-orientation fiber structures are typically achieved through high-speed spinning combined with subsequent thermal drawing. During the two-step melt spinning process, post-drawing induced alterations in the fiber’s internal super-molecular structure. Simultaneously, heat treatment promoted internal stress relaxation and continuous refinement of the crystalline structure, thereby enhancing fiber performance. Consequently, post-drawing is essential for fibers, and spinning speed plays a critical role in determining the post-drawn fiber properties.

Therefore, this work aims to investigate the influence of spinning processes on the properties and structure of PBS fibers, as well as the corresponding regulation mechanisms. First, the effects of spinning temperature and spinning speed on the properties and structure of PBS pre-oriented yarns were systematically investigated to determine the optimal spinning parameters. Subsequently, the evolution of properties and structures of pre-oriented yarns under maximum drawing conditions across different spinning speed systems was examined. Through simultaneous analysis of the correlations among mechanical properties (breaking strength, elongation at break), thermal behavior (melting enthalpy) and condensed state structures, this work reveals the structural performance regulation mechanism under the synergistic control of spinning–drawing processes.

## 2. Experiment Section

### 2.1. Materials

PBS pellets (trade name as TH803s, Mw = 89,331 g/mol, Mn = 41,740 g/mol, PDI = 2.14, MFR = 38.6 g/10 min (190 °C/2160 g)) were provided by Xinjiang Blueridge Tunhe Chemical Industry Co., Ltd., Changji, China.

### 2.2. Preparation of PBS Pre-Oriented Yarns and Investigation of Spinning Processes

**Figure 1:**
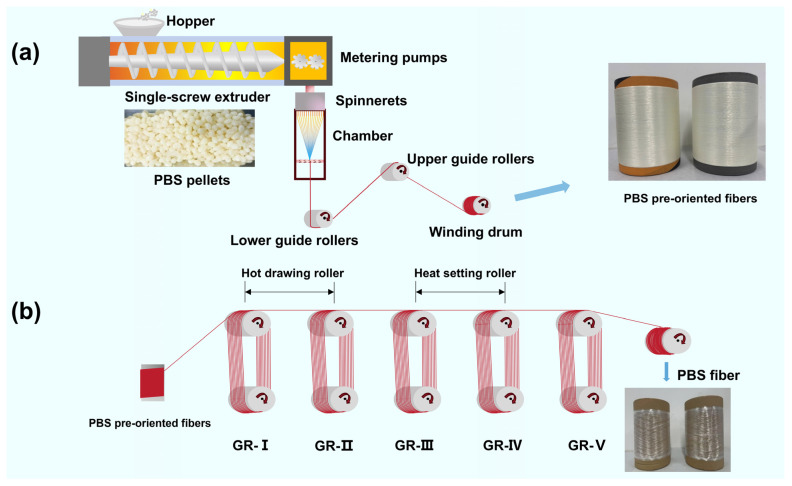
Flowchart of PBS fiber preparation: (**a**) melt spinning and (**b**) the subsequent drawing process of PBS pre-oriented yarns.

(1) The PBS chips were dried in a nitrogen drying oven at 100 °C for 6 h. Subsequently, the dried PBS chips were subjected to melt spinning using a homemade spinning device. A circular spinneret with a diameter of 0.18 mm and a length-to-diameter ratio of 0.54/0.18 was selected for the spinning assembly. The screw rotation speed was set to 18 rad/min, while the metering pump parameters were configured to 1.2 cc/rad with a rotation speed of 8 rad/min. The temperature settings for each zone of the spinning machine are listed in Table 1. The spinning speed (winding drum speed) was initially set to 2500 m/min. By adjusting the temperature of the spinning assembly zone to 195 °C, 205 °C and 215 °C, respectively, three types of pre-oriented fibers with varying spinning temperatures were obtained. This process aimed to investigate the influence of spinning temperature on PBS pre-oriented fibers. The spinning procedure is illustrated in Figure 1a.
(2) The spinning assembly zone temperature was controlled at 195 °C, and melt spinning was performed by varying the spinning speeds of 1200, 1500, 2000, 2300 and 2500 m/min to produce five pre-oriented yarns (POY) with different spinning speeds. This study investigated the effect of spinning speed on PBS pre-oriented yarns.
(3) The study investigates the maximum thermal drawing ratio of PBS-POY under five different spinning speeds, explores the influence of spinning speed on the final condensed state structure and properties of fibers and reveals the regulation mechanisms of fiber structure and performance during the spinning–drawing process. The drawing process is illustrated in Figure 1b, where GR-I and GR-II control the fiber’s drawing temperature, while GR-III and GR-IV regulate the heat-setting temperature of the fiber. The draw ratio is achieved through the speed ratio between GR-II and GR-III. The drawing temperature was set at 40 °C and the heat-setting temperature at 80 °C (detailed explanations regarding the selection of drawing parameters can be found in the Supplementary Materials).

### 2.3. Characterization

#### 2.3.1. Mechanical Properties Testing of PBS Fibers

The mechanical properties of the fiber multifilament were tested using a YG029A fully automatic single-yarn strength tester produced by Changzhou Dahua Electronic Instrument Co., Ltd. (Changzhou, China). According to the national standard GB/T 14344-2022 test method for tensile properties of chemical fiber filaments [40], the pretension was set to 0.05 cN/dtex, and the force drop was set to 10%. For fiber samples with an average elongation greater than 50%, the gauge length was set to 250 mm and the tensile speed was set to 250 mm/min. For fiber samples with an average elongation of less than 50%, the gauge length was set to 500 mm and the tensile speed was set to 500 mm/min. Ten sets of data were measured for each sample, and the average value was taken.

#### 2.3.2. Thermal Properties Testing of PBS Fibers

The crystallinity of the fibers was tested using a DSC250 differential scanning calorimeter produced by TA Instruments, New Castle, DE, USA. A fiber sample weighing 5–10 mg was placed in an aluminum crucible, and the test atmosphere was set to N2. The temperature was increased from 30 °C to 180 °C at a rate of 10 °C/min. The formula for calculating crystallinity is as follows:

(1) $$X_{c}=\frac{{\Delta H}_{m}}{{\Delta H}_{m}^{0}}\;\times \;100\%$$

Here, X_c_ and ΔH_m_ represent the crystallinity and melting enthalpy of the PBS fibers, respectively, and Δ$H_{m}^{0}$ is the theoretical melting enthalpy of PBS, taken as 110.5 J/g [34].

#### 2.3.3. WAXS

The 2D-WAXS data of the fibers were collected using a Xenocs Xeuss 3.0 system from Grenoble France. The specific testing conditions were as follows: copper palladium 8.05 KeV X-ray, an incident wavelength of 1.54189 Å, a detector-to-sample distance of 50 mm, a scanning angle range of 1–52° and an exposure time of 600 s for each fiber sample. The obtained WAXS data were analyzed using Fit2D V12.077 and Jade 6 software. Based on Bragg’s Equation (2) and Formulas (3)–(5) [41,42], the interplanar spacing (d_hkl_), crystallite size (D_hkl_), crystallinity (X_c_) and orientation degrees of the crystalline and amorphous regions of the PBS fibers were calculated.

(2) $$d_{\mathrm{hkl}}=\frac{\lambda }{{2\sin\theta }_{\mathrm{hkl}}\;\;}$$

(3) $$D_{\mathrm{hkl}}=\frac{K\lambda }{\left(\beta_{\mathrm{hkl}}{\;\times \;\cos\theta }_{\mathrm{hkl}}\right)}$$

(4) $$X_{c}=\frac{S_{c}}{S_{c}{\;+\;S}_{a}}\;\text{×}\;100\%$$

(5) $$f=\frac{180-H}{180\text{°}}\times 100\%$$

Here, K is the Scherrer constant, taken as 0.89, λ is the X-ray wavelength, taken as 1.54189 Å, β_hkl_ is the full width at half maximum (FWHM) of the diffraction peak, S_c_ is the integrated intensity of the crystalline region, S_a_ is the integrated intensity of the amorphous region, H is the FWHM at the azimuthal diffraction peak and f is the degree of orientation.

#### 2.3.4. SAXS

The 2D-SAXS data of the fibers were collected using a Xenocs Xeuss 3.0 system from France. The specific testing conditions were as follows: copper palladium 8.05 KeV X-ray, an incident wavelength of 1.54189 Å, a detector-to-sample distance of 1000 mm and an exposure time of 10 min. The two-dimensional color maps were integrated using Fit2D, and the thicknesses of the amorphous and crystalline regions as well as the long period were calculated using the one-dimensional electron density correlation function [43], as shown below:

(6) $$\frac{\int_{0}^{\infty }I\left(q\right)\cos\left(\mathrm{qz}\right)\mathrm{dq}}{\int_{0}^{\infty }I\left(q\right)\mathrm{dq}}$$

Here, z represents the distance in the direction perpendicular to the lamellar surface.

## 3. Result and Discussion

### 3.1. The Influence of Spinning Temperature on the Properties and Structure of PBS Pre-Oriented Yarns

In the spinning process, the spinning temperature is directly related to the flow properties of the melt. Flow instabilities not only impair the drawn structure and performance of fibers but may even completely hinder melt spinning. Excessively high spinning temperatures may induce thermal degradation of polymer chains, thereby reducing the mechanical properties of fibers. Therefore, precise control of the spinning temperature constitutes a crucial factor in producing high-strength fibers.

**Figure 2:**
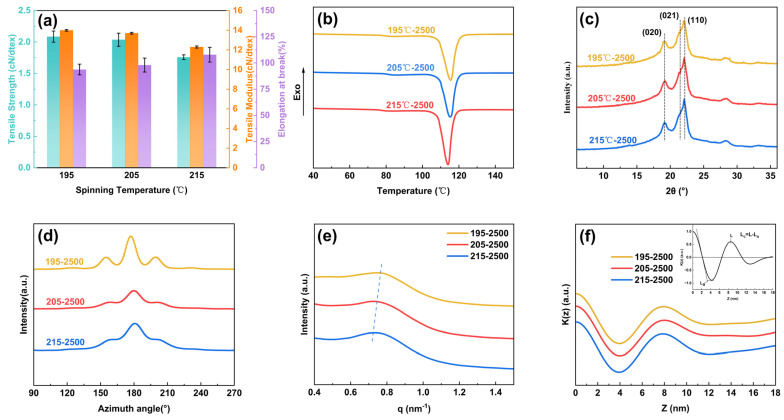
The performance and condensed state structure of PBS-POY under different spinning temperatures: (**a**) mechanical properties, (**b**) DSC curves at a heating rate of 10 °C/min, (**c**) 1D-WAXS curves, (**d**) azimuthal integration curves, (**e**) 1D-SAXS curves and (**f**) one-dimensional electron density correlation function K(z).

As shown in Figure 2a, the mechanical properties of PBS-POY under different spinning temperatures were presented in the bar chart. It can be observed that as the spinning temperature increased, the tensile strength of the pre-oriented yarns gradually decreased from 2.09 cN/dtex to 1.76 cN/dtex, while the tensile modulus progressively declined from 13.99 cN/dtex to 12.30 cN/dtex. Meanwhile, the elongation at break slightly increased from 93.8% to 107.9%. It can be concluded from Figure 2b that with the increase of spinning temperature, the melting enthalpy of PBS-POY gradually decreases and the crystallinity gradually decreases from 60.48% to 55.67%. (Table S7).

WAXS and SAXS were employed to characterize the crystalline structure changes of pre-oriented yarns under different spinning temperatures to elucidate the variations in mechanical properties and crystallinity of PBS-POY (Figure S5). As shown in Figure 2c, the 1D-WAXS curves of PBS-POY demonstrated that all fibers maintain the α-crystal form, with characteristic peaks consistently appearing at 2θ = 19.2°, 21.4° and 22.1°, corresponding to the (020), (021) and (110) crystal planes, respectively [44]. The crystal plane spacing, crystallite size and crystallinity calculated through peak deconvolution fitting using Origin 2024b software are listed in Table 2. As shown in the table, the crystallinity of PBS-POY gradually decreased from 64.27% to 59.23% with increasing spinning temperature, which aligned with the trend observed in DSC results. This indicated that excessively high spinning temperatures were unfavorable for fiber crystallization.

As the spinning temperature increased, the crystallite sizes on various crystal planes increased to different extents, particularly for the (021) crystal plane where the size grew from 9.267 nm to 9.311 nm. This phenomenon could be attributed to the reduced nucleation density in the amorphous regions of fibers and the extended cooling time to room temperature at higher spinning temperatures, which allowed molecular chains more adequate time for orderly arrangement. Consequently, this promoted crystallite growth and facilitated the formation of large spherulites within fibers. However, these large spherulites contain numerous internal defects that readily induced stress concentration during stretching, ultimately leading to a gradual reduction in the mechanical strength of the fibers. The interplanar spacing changed insignificantly with the increasing spinning temperature. The spacing on the (020) crystal plane slightly decreased from 2.678 nm to 2.673 nm, while other interplanar spacings remained essentially unchanged. This can be attributed to enhanced molecular chain mobility at elevated temperatures, where molecular chains were more prone to sliding under stress, leading to a slight contraction of interplanar spacing. As shown in Figure 2d (Table 2), the orientation degrees of different PBS-POY samples were calculated. At a spinning temperature of 195 °C, the crystalline region orientation degree reached 91.42% and the amorphous region orientation degree reached 83.7%. However, with increasing spinning temperatures, the crystalline region and amorphous region orientation degrees decreased to 89.45% and 82.41%, respectively. This phenomenon may have been caused by thermal degradation of molecular chains under high temperature conditions, which prevented the molecular chains from achieving effective preferential alignment along the external stress direction. Additionally, the molecular chains exhibited intense thermal motion, with chain disorientation becoming the dominant process. Consequently, under the combined effects of crystallization and orientation, the mechanical properties of PBS-POY progressively declined as the spinning temperatures increased.

SAXS can be used to characterize the macromolecular chain structures with dimensions ranging from 1 to 100 nm. Figure 2e showed the 1D-SAXS curves of PBS-POY at different spinning temperatures, where distinct scattering peaks were observed in the scattering vector range of 0.6–0.9 nm^−1^, indicating the presence of a long-period structure in the PBS-POY structure. As the spinning temperature increased, the long period of the fibers increased from 7.80 nm to 8.05 nm. Through calculations using the one-dimensional electron density correlation function shown in Figure 2f, it was revealed that with increasing spinning temperatures, the amorphous layer thickness of PBS-POY decreased from 3.12 nm to 3.10 nm, while the crystalline layer thickness increased from 4.68 nm to 4.95 nm (Table S8); this led to a consequent rise in the long period of the fibers.

### 3.2. The Influence of Spinning Speeds on the Properties and Structure of PBS Pre-Oriented Yarns

**Figure 3:**
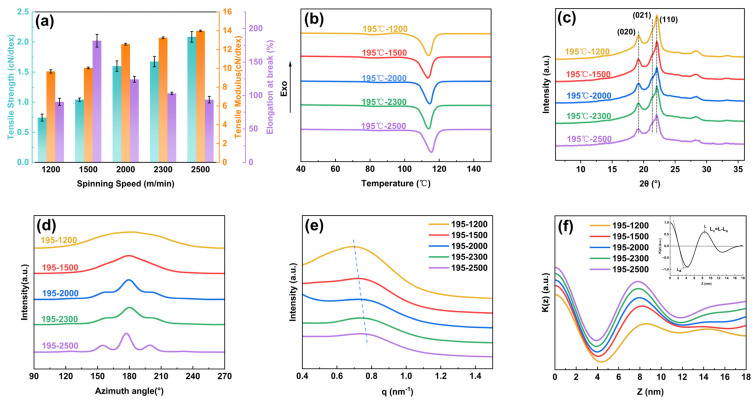
The performance and condensed state structure of PBS-POY under different spinning speeds: (**a**) mechanical properties, (**b**) DSC curves at a heating rate of 10 °C/min, (**c**) 1D-WAXS curves, (**d**) azimuthal integration curves, (**e**) 1D-SAXS curves and (**f**) one-dimensional electron density correlation function K(z).

As shown in Figure 3a, the mechanical strength of PBS-POY significantly increased from 0.74 cN/dtex to 2.09 cN/dtex as the spinning speeds rose from 1200 m/min to 2500 m/min, while the tensile modulus increased from 9.68 cN/dtex to 13.99 cN/dtex. Notably, when the spinning speed exceeded 1500 m/min, the elongation at break gradually decreased with increasing spinning speed. However, the fiber sample spun at 1200 m/min exhibiting an elongation at break of only 90.5% (Table S9). As shown in Figure 3b, the DSC results revealed that the enthalpy of fusion (ΔH) of PBS-POY increased from 61.11 J/g to 66.83 J/g with the elevation of spinning speed, accompanied by a shift of the onset melting temperature toward higher temperatures. Further calculations using the crystallinity formula demonstrated that the crystallinity rose from 55.30% to 60.48%.

**Figure 4:**
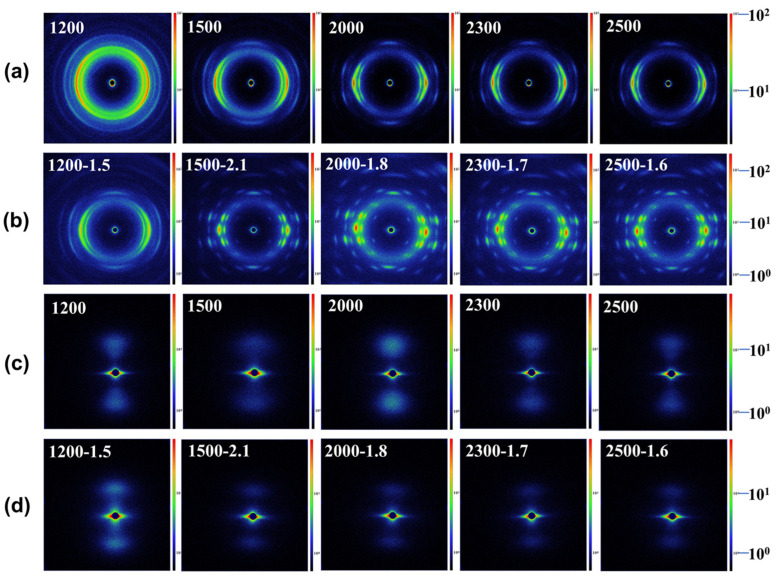
2D-WAXS and SAXS images of fibers at different spinning speeds: (**a**,**c**) before drawing, (**b**,**d**) after drawing.

The crystalline structure of PBS-POY fibers with different spinning speeds was characterized by WAXS and the two-dimensional (2D) images was shown in Figure 4a. As shown in Figure 3c, all fibers exhibited the α-crystal form, with characteristic peaks appearing near 2θ = 19.2°, 21.4° and 22.1°, corresponding to the (020), (021) and (110) crystal planes, respectively. The interplanar spacing, crystallite size and crystallinity calculated using relevant formulas are listed in Table 3. The results demonstrated that increasing spinning speeds induced a progressive enhancement in PBS-POY crystallinity from 55.30% to 60.48%, aligning with the DSC trend. This confirmed that higher spinning speeds accelerated crystallization, improved the crystal structure and boosted the fiber’s thermal stability, ultimately shifting the onset melting temperature to a higher range. While the interplanar spacing remained largely unchanged, the crystallite size generally decreased with increasing spinning speed, particularly for the (020) plane (Table 3), where the crystallite size decreased from 11.591 nm (SS = 1200) to 9.267 nm (SS = 2500). These phenomena can be attributed to intensified chain packing under higher spinning stresses, which induced nucleation and generated more small crystallites. These smaller crystallites act as “physical crosslinking points,” restricting chain mobility under stress and thereby increasing the modulus while reducing the elongation at break of PBS-POY. The investigation of spinning speed effects on fiber orientation (Figure 3d, Table 3) demonstrated that both crystalline and amorphous orientations of PBS-POY increased with spinning speed, reaching maximum values of 91.42% and 83.7%, respectively, at 2500 m/min. This enhanced orientation in both crystalline and amorphous regions during fiber formation contributes to the improved mechanical properties of the pre-oriented yarns.

The 1D-SAXS curves and 2D-SAXS images corresponding to fibers produced at various spinning speeds are presented in Figure 3e and Figure 4c, respectively. It was evident that the q-value of PBS-POY increased with higher spinning speeds, indicating a decrease in the long period as the spinning speed increased. When the spinning speed reached 2500 m/min, the long period of PBS-POY decreased from 8.30 nm to 7.80 nm. As shown by the one-dimensional electron density correlation function calculations in Figure 3f, both the crystalline thickness and amorphous thickness of PBS-POY decreased with increasing spinning speed—from 5.23 nm to 4.68 nm for the crystalline region and from 3.37 nm to 3.12 nm for the amorphous region (Table S10). This was because when the spinning speed increased, the fiber diameter became finer, the fibers were rapidly cooled upon extrusion from the spinneret, which led to the rapid “freezing” of molecular chains. This dynamic ultimately shortened the crystal growth time and reduced the thickness of crystal domains. Simultaneously, the high spinning speeds induced strong molecular chain orientation, potentially triggering stress-induced crystallization. This promoted the transformation of molecular chains from the amorphous region to the crystalline region, resulting in a decrease in amorphous thickness. These findings further confirmed that increasing spinning speed enhanced the stacking of molecular chains in both the crystalline and amorphous regions of PBS-POY.

### 3.3. Effect of Spinning Speed on the Properties and Structure of Fibers After Maximum Drawing

As a core control parameter in the spinning process, spinning speed played a decisive role in the formation of the condensed state structure of pre-oriented yarns. Pre-oriented yarns prepared at different spinning speeds develop gradient differences in the orientation state and crystalline structure internally. These structural characteristics directly determine the regulation window for subsequent drawing processes, particularly manifesting in the critical indicator of maximum draw ratio. Generally, higher spinning speeds resulted in greater orientation degrees of pre-oriented yarns and correspondingly smaller maximum draw ratios. Notably, WAXS characterization in preliminary studies revealed that increased spinning speeds effectively refine the crystalline grain size. This nanoscale structural modulation exerts a significant influence on the structural evolution induced by drawing processes.

**Figure 5:**
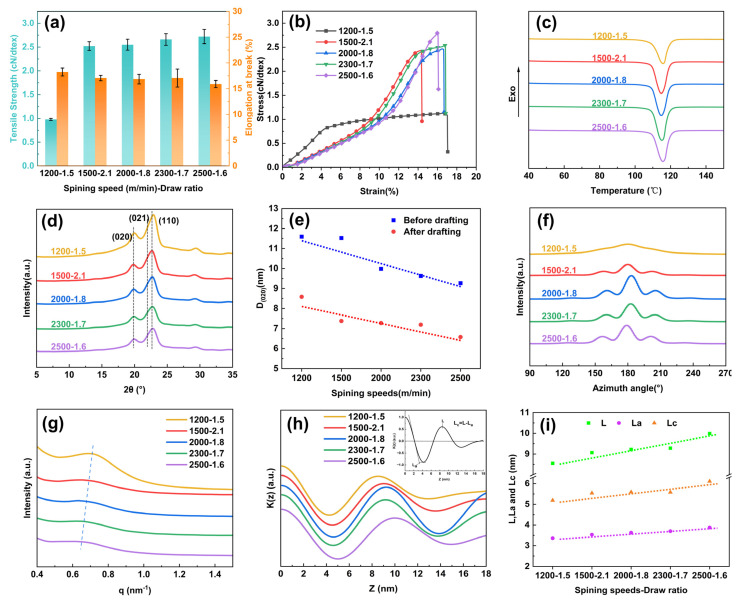
Properties and condensed state structure of PBS-POY after drawing at maximum draw ratio with different spinning speeds: (**a**) mechanical properties, (**b**) tensile stress–strain curves, (**c**) DSC curves at a heating rate of 10 °C/min, (**d**) 1D-WAXS curves, (**e**) changes in the grain size of the (020) crystallographic plane of PBS-POY with different spinning speeds before and after drawing, (**f**) azimuthal integration curves, (**g**) 1D-SAXS curves, (**h**) one-dimensional electron density correlation function K(z) and (**i**) variation of long period, thickness of crystalline and amorphous region.

As shown in Figure 5a,b, the tensile strength of the drawn fibers continuously increased with higher spinning speeds. When the spinning speed reached 2500 m/min, the fiber’s breaking strength rose from 0.98 cN/dtex to 2.72 cN/dtex. Furthermore, the DSC results in Figure 5c revealed that the enthalpy of fusion of the fibers after maximum draw ratio stretching gradually increased from 62.73 J/g to 78.52 J/g with elevated spinning speeds, corresponding to a crystallinity increase from 56.77% to 71.06% (Figure 5c, Table S11). Compared to the undrawn PBS-POY fibers, the drawn fibers exhibited significantly enhanced mechanical strength, melting enthalpy and crystallinity.

The differences in the mechanical properties and crystallinity of the final fibers under different spinning speeds were explained by determining the crystal structure and orientation. Figure 4b showed the 2D-WAXS image of the fiber after drawing. As calculated from the 1D-WAXS curves in Figure 5d, the crystallinity of the drawn fibers increased from 61.7% to 71.58% with the rise in spinning speeds, consistent with the DSC crystallinity results. The crystal sizes on various crystal planes of the drawn fibers were significantly reduced compared to those before drawing. For instance, the crystal size on the (020) plane of the fiber sample spun at 1500 m/min decreased from 11.521 nm to 7.276 nm after drawing (Figure 5e). Furthermore, the crystal sizes on all crystal planes of the drawn fibers exhibited a decreasing trend with increasing spinning speeds. This indicated that higher spinning speeds facilitated the reduction of the average crystal size in the final fibers. Through the calculation of orientation degree from Figure 5d, it was observed that both crystalline and amorphous region orientation degrees of the final fibers showed an increasing trend with the elevation of spinning speed. Specifically, the crystalline orientation increased from 85.23% to 95.95%, while the amorphous orientation rose from 77.02% to 92.29%, indicating that higher spinning speeds facilitated orientation enhancement in the final fibers. Under the synergistic effects of simultaneous improvements in crystallinity and orientation, the molecular chain alignment became optimized and intermolecular forces were strengthened. Consequently, both mechanical properties and thermal performance of the final fibers were significantly enhanced with increased spinning speed. Based on mechanical property analysis and WAXS results, it was speculated that the structural and performance variations in the final fibers were associated with the structural evolution of spherulites during the spinning–drawing process.

**Figure 6:**
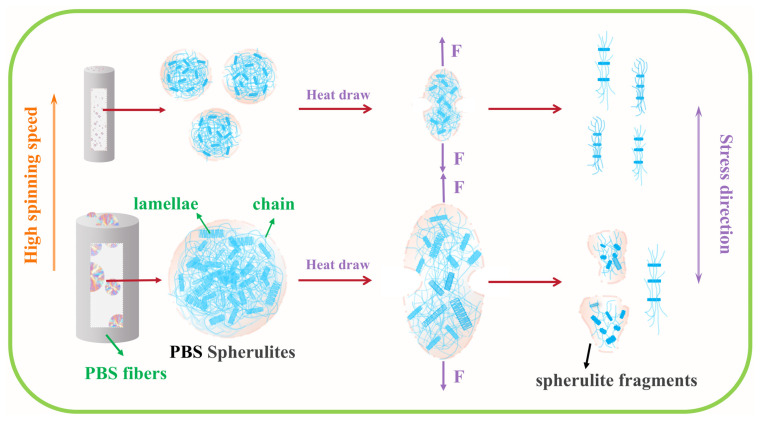
Schematic of stress-induced spherulite structural evolution during drawing.

As a semi-crystalline polymer, PBS tended to form large spherulites during the cooling process. When the spinning speeds were low, the nascent fiber was subjected to relatively small external stress and nucleation was hindered. Under these conditions, the crystal grains continuously grew and branched to form more lamellae, ultimately resulting in the formation of large and fewer spherulites within the fiber. These large spherulites contained numerous molecular chains with a disordered arrangement embedded within them, which restricted the movement of the molecular chains. As the spinning speeds increased, the fiber experienced a higher tensile stress, causing it to be rapidly drawn and thinned while the cooling rate intensified [45]. The tensile stress induced the formation of crystal nuclei, leading to a gradual increase in the number of spherulites within the fiber and a simultaneous reduction in their size. When the pre-oriented fiber was stretched, the spherulites first began to deform along the stress direction. During this stage, the stress caused molecular chains within the spherulite structure to align along the deformation direction, while sliding between lamellae was initiated. As a result, the spherulites were gradually stretched and separated [46]. As the stress continued to increase, the spherulites underwent slip orientation and separation of lamellae until all lamellae within the spherulites were aligned with their long-period directions nearly parallel to the deformation direction. At this stage, further orientation of the crystals became extremely difficult, and the spherulitic structure gradually diminished [47]. These aligned small lamellae acted as physical crosslinking points, which resulted in a continuous increase in the fiber’s mechanical strength (as shown in Figure 6). This phenomenon of spherulites being “torn” into smaller lamellae manifested as smaller spherulite dimensions correlating with more uniform tearing. The fiber sample spun at 1200 m/min exhibited a lower draw ratio and inferior mechanical strength, which was likely attributed to excessively large spherulites formed during PBS fiber formation. These large spherulites contained internal defects that induced localized stress concentration during stretching, triggering partial molecular chain scission within the spherulites. Consequently, the spherulitic structure failed to separate uniformly into lamellae but instead fragmented into irregularly sized spherulitic debris. These fragments restricted the aligned arrangement of molecular chains, thereby compromising the fiber’s mechanical performance.

Figure 5e displayed the 1D-SAXS curve characteristics of PBS pre-oriented yarns prepared at different spinning speeds after maximum ratio drawing. The experimental results indicated that all samples exhibited scattering peaks within the range of 0.5–0.8 nm^−1^, demonstrating that the fibers maintained a distinct long-period ordered structure even after ultimate drawing. Notably, the scattering vector (q) corresponding to the maximum scattering peak showed a systematic decrease with increasing spinning speeds, revealing a positive correlation between the final fiber’s long period and spinning speeds. As revealed by the one-dimensional electron density correlation function calculations in Figure 5h, and Table 4, the long period of the final fibers significantly expanded from 8.55 nm to 9.99 nm as spinning speed increased. Specifically, the amorphous layer thickness increased from 3.36 nm to 3.88 nm, while the crystalline layer thickness showed more pronounced growth from 5.19 nm to 6.11 nm, indicating that crystalline thickening dominated the long-period expansion (Figure 5i, Table S12). This phenomenon can be attributed to the thermal drawing process where thinner lamellae melt and molecular chains reorganize, leading to amorphous layer thickening. Concurrently, the high-stress drawing field drives orientation-induced crystallization of molecular chains in amorphous regions, facilitating their transformation into crystalline phases and consequently enhancing crystalline thickness. Importantly, the enhanced initial structural regularity achieved through higher spinning speeds requires greater stress application during subsequent drawing to overcome molecular chain motion barriers. This intensified stress field not only accelerated the dynamic equilibrium between thin crystal melting and recrystallization but also generated stronger orientation driving forces at amorphous–crystalline interfaces. Consequently, more significant stress-induced crystallization effects emerge in high-speed spinning systems, ultimately resulting in an increased long period with elevated spinning speeds.

## 4. Conclusions

PBS pre-oriented yarns with different spinning parameters were prepared via melt spinning, and the effects of spinning temperature and speed on the fiber properties and structure were systematically investigated. The results indicated that increasing the spinning temperature suppressed the orientation and crystallization of the pre-oriented yarns. Higher spinning speeds reduced the crystal size of PBS pre-oriented yarns, with the long period decreasing from 8.6 nm to 7.8 nm, accompanied by tighter and more ordered molecular chain packing. This structural evolution led to an increase in tensile strength from 0.74 cN/dtex to 2.09 cN/dtex. Furthermore, spinning speed significantly influenced the properties and structure of the drawn fibers. Elevated spinning speeds promoted the formation of more numerous and smaller spherulites during fiber formation. These smaller spherulites were uniformly split into oriented lamellae along the stress direction during hot drawing, resulting in reduced average crystal size, enhanced crystallinity, improved orientation and ultimately increased mechanical strength up to 2.72 cN/dtex. SAXS analysis revealed that the crystalline layer thickness in final fibers increased from 5.19 nm to 6.11 nm with higher spinning speeds, demonstrating that crystal thickening dominated the enlargement of the long period.

## Figures and Tables

**Table 1:** Temperatures of different zones in the spinning machine.

Zones	Zone 1	Zone 2	Zone 3	Zone 4	Spinning Pack
Temperature °C	160	180	190	195	195

**Table 2:** The condensed state structure of PBS pre-oriented yarns at different spinning temperatures.

ST (°C)	Crystal Grain Size (nm)			Interplanar Spacing (nm)			Fc (%)	Fa (%)	Xc (%)	Single Fiber Diameter (um)
	020	021	110	020	021	110				
195	9.267	6.042	10.037	2.678	2.397	2.319	91.42	83.7	64.27	12.06
205	9.311	6.077	10.345	2.673	2.397	2.318	90.68	76.71	62.94	12.04
215	9.349	6.116	10.348	2.673	2.397	2.318	89.45	82.41	59.23	12.05

**Table 3:** The condensed state structure of PBS-POY at different spinning speeds.

SS (m/min)	Crystal Grain Size (nm)			Interplanar Spacing (nm)			Fc (%)	Fa (%)	Xc (%)	Single Fiber Diameter (um)
	020	021	110	020	021	110				
1200	11.591	6.412	10.732	2.668	2.394	2.316	71.84	51.32	58.69	18.24
1500	11.521	6.311	11.726	2.671	2.393	2.317	83.62	69.57	59.95	16.81
2000	9.984	6.253	10.205	2.672	2.399	2.318	90.19	82.65	61.11	13.62
2300	9.629	5.932	10.084	2.671	2.398	2.316	90.95	83.55	62.05	12.45
2500	9.267	6.042	10.037	2.678	2.397	2.319	91.42	83.7	64.27	12.06

**Table 4:** The condensed state structure of PBS-POY at maximum draw ratio for different spinning speeds.

SS-DR	Crystal Grain Size (nm)			Interplanar Spacing (nm)			Fc (%)	Fa (%)	Xc (%)	Single Fiber Diameter (um)
	020	021	110	020	021	110				
1200-1.5	8.583	6.063	9.332	2.571	2.307	2.230	85.23	77.02	61.17	15.58
1500-2.1	7.378	5.787	8.690	2.590	2.318	2.248	95.22	91.01	67.07	11.70
2000-1.8	7.276	5.770	7.668	2.588	2.321	2.249	95.76	91.44	68.54	11.40
2300-1.7	7.191	5.901	8.232	2.580	2.311	2.240	95.7	91.81	66.33	10.95
2500-1.6	6.576	5.757	8.528	2.569	2.297	2.231	95.98	92.29	71.58	10.29

## Data Availability

The original contributions presented in this study are included in the article. Further inquiries can be directed to the corresponding authors.

## Supplementary Materials

The following supporting information can be downloaded at: https://www.mdpi.com/article/10.3390/polym17091138/s1, Figure S1. Properties and microstructure of fibers at different drawing temperatures. (a) Mechanical properties, (b) DSC curves at a heating rate of 10 °C/min, (c) 1D-WAXS curves and (d) azimuthal integration curves; Figure S2. Properties and microstructure of fibers at different heat-setting temperatures: (a) mechanical properties, (b) DSC curves at a heating rate of 10 °C/min, (c) 1D-WAXS curves and (d) azimuthal integration curves; Figure S3. The properties and microstructure of fibers under different draw ratios. (a) Mechanical properties, (b) DSC curves at a heating rate of 10 °C/min, (c) 1D-WAXS curves and (d) azimuthal integration curves; Figure S4. 2D-WAXS images of fibers under different drawing processes. (a) Different drawing temperatures, (b) different heat-setting temperatures and (c) different draw ratios; Figure S5. 2D-WAXS and SAXS images of PBS-POY at different spinning temperatures. (a)WAXS and (b)SAXS; Figure S6. TGA curve of PBS spinning raw material; Table S1. Mechanical properties and crystallinity of PBS fibers at different drawing temperatures; Table S2. The condensed state structure of PBS fibers at different drawing temperatures; Table S3. Mechanical properties and crystallinity of PBS fibers at different heat setting temperatures; Table S4. The condensed state structure of PBS fibers at different heat setting temperatures; Table S5. Mechanical properties and crystallinity of PBS fibers at different draw ratios; Table S6. The condensed state structure of PBS fibers at different draw ratios; Table S7. Mechanical properties and crystallinity of PBS-POY at different spinning temperatures; Table S8. The microstructure of PBS-POY at different spinning temperatures; Table S9. Mechanical properties and crystallinity of PBS-POY at different spinning speeds; Table S10. The microstructure of PBS-POY at different spinning speeds; Table S11. Mechanical properties and crystallinity of PBS-POY at maximum draw ratio for different spinning speeds; Table S12. The microstructure of PBS-POY at maximum draw ratio for different spinning speeds; Table S13. Thermal stability parameters of PBS spinning raw material.
