# Supplementary material for: The Influence of Spinning Process on the Properties and Structure of PBS Fibers
Source: Polymers (Basel). 2025 Apr 22;17(9):1138. doi: 10.3390/polym17091138 (PMC12073709; doi:10.3390/polym17091138)
Supplement: Supplementary file 1 [file polymers-17-01138-s001.zip › polymers-3583303-supplementary.pdf]

1. The Influence of Drawing Process on Fiber Properties and Structure

In the process of melt spinning, the performance of pre-oriented fibers often did not meet the required standards, and post-drawing could alter the super-molecular structure within the fibers. Additionally, heat treatment allowed for the relaxation of internal stresses and the refinement of the crystalline structure, thereby improving the fiber's properties. Therefore, it is necessary to perform post-drawing on the fibers, and selecting appropriate process parameters is crucial for enhancing the performance of PBS fibers. To explore the optimal drawing process for the fibers, we conducted a systematic study using PBS fibers produced at a spinning speed of 1500 m/min. This study investigated the effects of drawing temperature, draw ratio and heat setting temperature on the fiber's properties and its condensed state structure. First, the heat setting temperature was set at 80°C and the draw ratio was fixed at two times. The drawing temperature was varied to 35°C, 40°C, 45°C, 50°C and 50°C to investigate the effect of drawing temperature on the fibers, thereby determining the optimal drawing temperature. Subsequently, under the optimal drawing temperature, the draw ratio was maintained at two times, and the heat setting temperature was adjusted to 60°C, 70°C, 80°C, 85°C and 90°C to study the influence of heat setting temperature and identify the optimal heat setting temperature. Finally, under the optimal drawing and heat setting temperatures, the draw ratio was varied to 1.4, 1.6, 1.8, 2.0 and 2.1 times to explore the effect of draw ratio on the fibers.

1.1 The influence of drawing temperatures on the properties and structure of fibers

The drawing temperatures is a significant factor influencing the properties of fibers after drawing. To investigate the impact of drawing temperature on the performance and structure of PBS fibers, we first measured the mechanical and thermal properties of fibers at different drawing temperatures. As shown in Figure S1a and S1b, it can be observed that as the drawing temperature increased to 40°C, the tensile strength of PBS fibers reached a maximum of 2.38 cN/dtex. At this point, the crystallinity measured by DSC also reached its highest value of 67.57%. However, as the drawing temperature continued to rise, both the tensile strength and crystallinity of the fibers decreased (Table S1).

To explain the changes in fiber properties, wide-angle X-ray scattering (WAXS) was employed to characterize the evolution of the condensed state structure of PBS fibers during the hot drawing process. As shown in Figure S1c, all PBS fibers exhibited the α-crystal form. When the drawing temperatures is 40°C, the crystallinity reached its maximum value of 69.17% (Table S2), and then decreased as the temperature increased, which is consistent with the DSC results. The grain sizes on the (021) and (110) crystal planes initially decreased and then increased, reaching their minimum values at a drawing temperature of 40°C. Interestingly, as calculated from Figure S1d, the orientation degrees of both the crystalline and amorphous regions of the fibers showed a trend of first increasing and then decreasing, reaching their maximum values of 95.02% and 90.43%, respectively, at 40°C. This may be attributed to the fact that when the drawing temperature was below 40°C, the molecular chains have limited mobility due to the low temperature. As the drawing temperature increased, the mobility of the molecular chains improved, making it easier for them to align regularly along the direction of the external force, thereby increasing the orientation degree. Highly oriented molecular chains can induce the formation of crystal nuclei, leading to an increase in the number of grains and a reduction in their average size; at this stage, the internal crystallization of the fibers became more refined. When the drawing temperatures exceeded 40°C, the molecular

chains primarily exhibited disorientation behavior, resulting in a decrease in the orientation degree. Consequently, the tensile strength of the fibers continuously decreased.

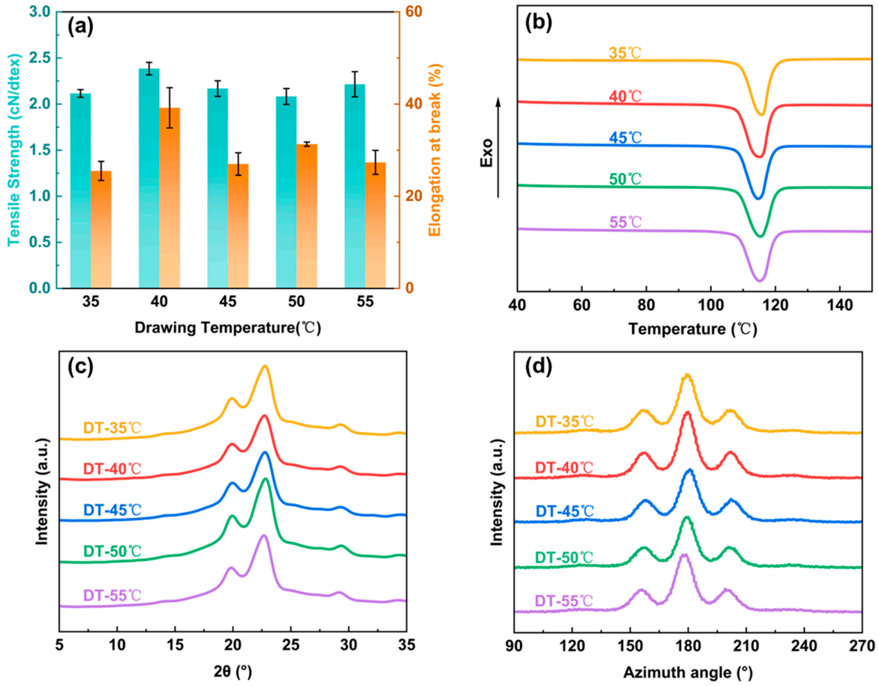

Figure S1. Properties and microstructure of fibers at different drawing temperatures. (**a**) Mechanical properties, (**b**) DSC curves at a heating rate of 10°C/min, (**c**) 1D-WAXS curves and (**d**) azimuthal integration curves.

Table S1. Mechanical properties and crystallinity of PBS fibers at different drawing temperatures.

DT(℃)	Linear density (dtex)	Breaking strength (cN/dtex)	SD	Elongation at break (%)	SD	Melting enthalpy (J/g)	Xc (%)
35	54.4	2.12	0.042	25.5	2.065	74.08	67.04
40	55.5	2.38	0.068	39.2	4.369	76.87	69.57
45	54.1	2.17	0.085	27.0	2.436	74.66	67.57
50	54.9	2.08	0.086	31.3	0.455	74.01	66.98
55	54.4	2.22	0.137	27.4	2.607	75.57	68.39

Table S2. The condensed state structure of PBS fibers at different drawing temperatures.

DT (℃)	Crystal grain size(nm)				Interplanar spacing(nm)				Fc(%)	Fa(%)	Xc(%)	Single fiber diameter(um)
	020	021	110	111	020	021	110	111				
35	7.886	5.736	8.741	6.875	2.579	2.311	2.236	1.760	94.48	89.61	66.42	11.81
40	7.509	5.489	7.881	7.041	2.579	2.307	2.237	1.763	95.02	90.43	69.14	11.83
45	7.347	5.920	7.654	6.318	2.573	2.309	2.236	1.761	94.64	90.14	68.69	11.80
50	8.574	6.400	8.658	7.898	2.574	2.309	2.233	1.757	94.86	89.05	67.63	11.82
55	7.504	5.837	8.385	7.032	2.587	2.319	2.246	1.767	94.43	88.87	68.08	11.83

1.2 The influence of heat-setting temperature on the properties and structure of fibers

After thermal drawing, the condensed state structure and mechanical strength of the fibers were improved. However, the internal structure of the fibers at this stage was unstable, necessitating heat setting to eliminate internal stresses and stabilize the oriented structure. As shown in Figure S2a, when the heat setting temperature is 80°C, the tensile strength of PBS fibers initially increased from 2.1 cN/dtex to 2.38 cN/dtex and then decreased to 2.11 cN/dtex as the temperature rose further. DSC results indicated that the

crystallinity first increased and then decreased, reaching a maximum value of 69.57% at 80°C (Table S3).

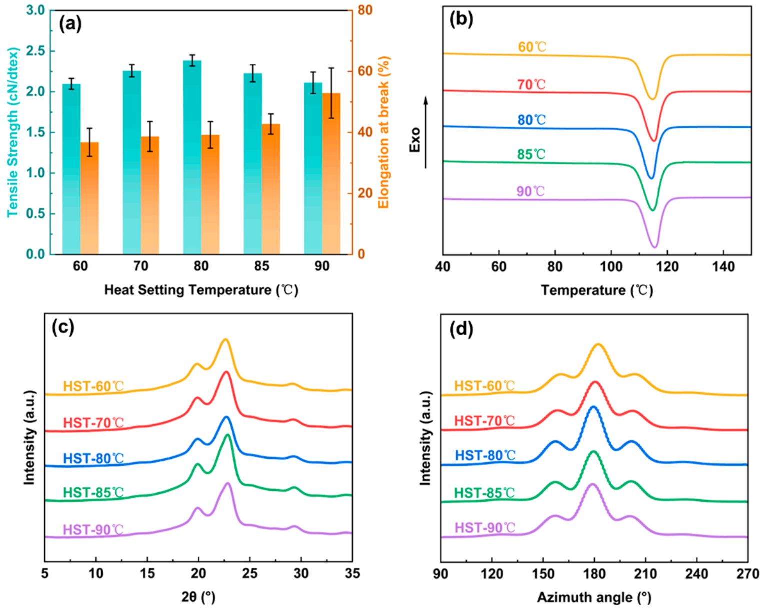

Figure S2. Properties and microstructure of fibers at different heat-setting temperatures: (**a**) mechanical properties, (**b**) DSC curves at a heating rate of 10°C/min, (**c**) 1D-WAXS curves and (**d**) azimuthal integration curves.

As calculated from the WAXS curves in Figure S2c, the crystallinity of PBS fibers reached a maximum value of 69.14% when the heat-setting temperature increased to 80°C. As the temperature further rose to 90°C, the crystallinity gradually decreased to 62.36%, which was consistent with the DSC results. This may be attributed to the fact that as the heat-setting temperature increased, the crystalline structure of PBS fibers gradually became more refined. However, a further rise in temperature could lead to excessive thermal motion of the molecular chains, thereby disrupting the crystalline structure. The interplanar spacings of (020), (110) and (111) decreased with the increase of heat-setting temperatures. Interestingly, the grain sizes of the (020) and (111) planes increased with rising heat setting temperature, growing from 7.338 nm and 6.347 nm to 8.633 nm and 7.511 nm, respectively (Table S4). This is due to grain growth under high-temperature conditions, which led to a gradual reduction in the distances between crystal planes. From Figure S2d, the orientation degrees of both the crystalline and amorphous regions of PBS fibers can be calculated. As shown in Table S4, when the heat-setting temperature was 80°C, the orientation degrees of both the crystalline and amorphous regions reached their maximum values. Then the orientation of the fibers decreased as the temperature continues to increase, because in a certain temperature range, the higher the heat-setting temperatures, the stronger the motility of the molecular chains, and the more conducive to the arrangement of the molecular chains along the axial orientation. When the temperature was excessively high, the disorientation effect of the molecular chains became predominant. Therefore, under the combined effects of crystallization and orientation, the tensile strength of the PBS fibers was maximized at a heat setting temperature of 80°C.

Table S3. Mechanical properties and crystallinity of PBS fibers at different heat setting temperatures.

HST(℃)	Linear density（dtex）	Breaking strength（cN/dtex）	SD	Elongation at break（%）	SD	Melting enthalpy（J/g）	Xc (%)
60	55.6	2.10	0.068	36.8	4.592	70.20	63.53
70	55.4	2.26	0.075	38.7	4.910	72.36	65.48
80	55.7	2.38	0.068	39.2	4.369	76.87	69.57
85	53.1	2.23	0.105	42.8	3.321	74.99	67.86
90	52.9	2.11	0.132	52.9	8.218	70.37	63.68

Table S4. The condensed state structure of PBS fibers at different heat setting temperatures.

HST(℃)	Crystal grain size(nm)				Interplanar spacing(nm)				Fc(%)	Fa(%)	Xc(%)	Single fiber diameter(um)
	020	021	110	111	020	021	110	111				
60	7.338	5.279	7.358	6.347	2.587	2.314	2.246	1.769	93.14	87.5	64.29	11.84
70	7.448	5.568	8.194	7.003	2.582	2.312	2.239	1.765	93.96	88.79	67.88	11.81
80	7.509	5.489	7.881	7.041	2.579	2.307	2.237	1.763	95.02	90.43	69.14	11.78
85	8.200	6.314	9.834	7.070	2.576	2.311	2.234	1.758	94.86	90.51	63.04	11.86
90	8.633	6.852	9.755	7.511	2.576	2.313	2.233	1.757	93.69	88.33	62.36	11.80

1.3 The influence of draw ratio on the properties and structure of fibers

To investigate the effects of drawing stress on the properties and crystal structure of PBS fibers, the mechanical and thermal properties were first tested at different draw ratios. As shown in Figure S3a, as the draw ratio increased to 2.1 times, the tensile strength of the fibers rose from 1.64 cN/dtex to 2.52 cN/dtex, while the elongation at break decreased from 93.5% to 17.1%. The DSC curves in Figure S3b revealed that the crystallinity of the fibers increased from 60.33% to 69.78% (Table S5).

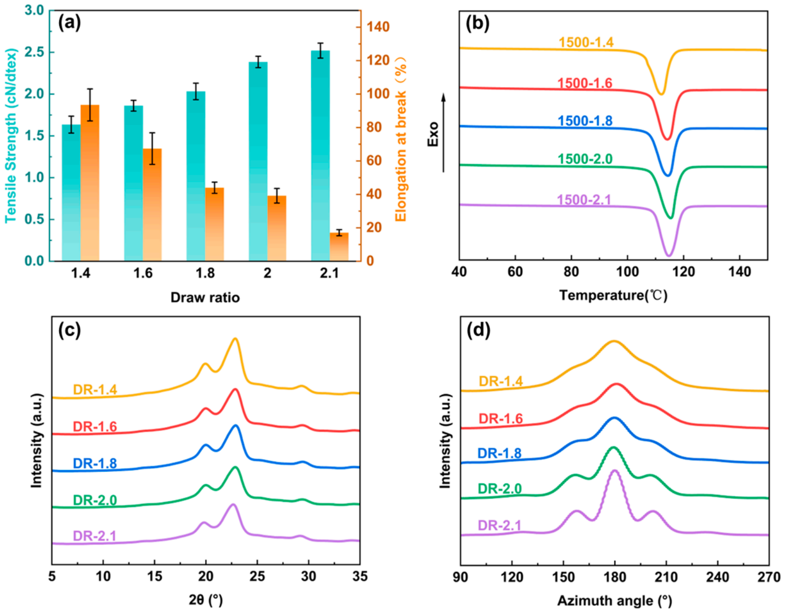

Figure S3. The properties and microstructure of fibers under different draw ratios. (**a**) Mechanical properties, (**b**) DSC curves at a heating rate of 10°C/min, (**c**) 1D-WAXS curves and (**d**) azimuthal integration curves.

As shown in the 1D-WAXS curves in Figure S3c, the crystallinity of the fibers increased from 60.26% to 67.07% as the draw ratio increased, which was consistent with

the trend observed in the previous DSC results, indicating that stress-induced crystallization occurred during the hot drawing process. The grain sizes generally showed a decreasing trend, particularly on the (020) and (111) crystal planes, where the grain sizes decreased from 8.38 nm and 7.49 nm to 7.378 nm and 6.607 nm, respectively, as the draw ratio increased (Table S6). This may be attributed to the increased stress promoting the formation of smaller grains, which acted as "physical crosslinking points," restricting the movement of molecular chains, so the elongation at break is constantly decreasing. The interplanar spacing changed were relatively minor; the interplanar spacing of the (020) crystal plane initially decreased from 2.573 nm to 2.567 nm as the draw ratio increased, and then subsequently increased to 2.59 nm. The interplanar spacing of the (111) crystal plane reached a minimum of 1.753 nm when the draw ratio increased to two times, and then subsequently increased to 1.765 nm (Table S6), this could be due to the sliding between lamellae, leading to a reduction in interplanar spacing. From the azimuthal integration curves in Figure S3d, it can be calculated that as the draw ratio increased, the orientation degree of the crystalline region rose from 85.46% to 95.22%, and that of the amorphous region increased from 78.76% to 90.99% (Table S6). This indicated that external stress promoted the orientation of both crystalline and amorphous regions, resulting in more regular alignment of molecular chains along the fiber axis and enhancing the tensile strength of the fibers.

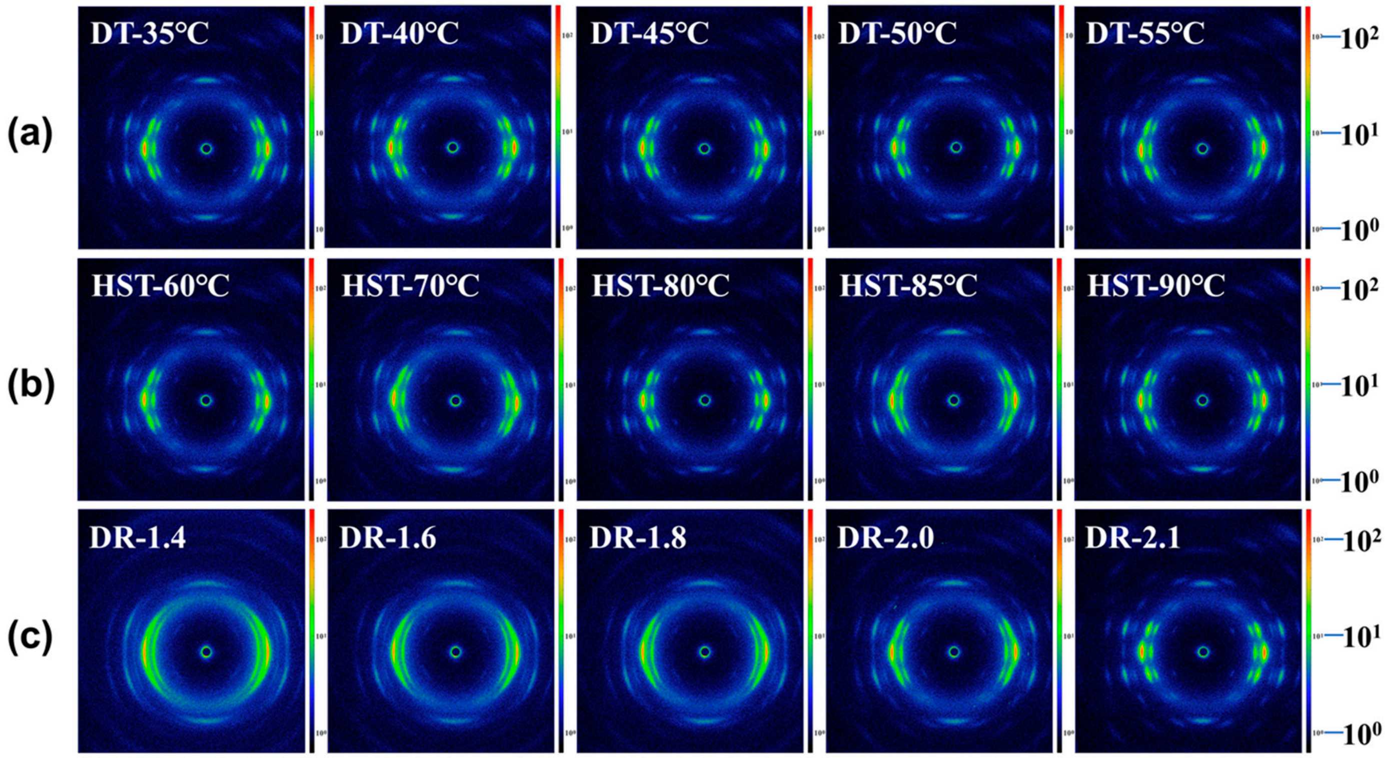

Figure S4. 2D-WAXS images of fibers under different drawing processes. (**a**) Different drawing temperatures, (**b**) different heat-setting temperatures and (**c**) different draw ratios.

Table S5. Mechanical properties and crystallinity of PBS fibers at different draw ratios.

Draw ratio	Linear density (dtex)	Breaking strength (cN/dtex)	SD	Elongation at break (%)	SD	Melting enthalpy (J/g)	Xc (%)
1.4	69.2	1.64	0.101	93.5	9.580	66.66	60.33
1.6	61.4	1.86	0.064	67.4	9.459	72.45	65.57
1.8	58.7	2.03	0.099	40.1	3.343	74.22	67.17
2.0	55.7	2.38	0.068	39.2	4.369	76.87	69.57
2.1	51.4	2.52	0.090	17.1	1.826	77.11	69.78

Table S6. The condensed state structure of PBS fibers at different draw ratios.

Draw ratio	Crystal grain size(nm)				Interplanar spacing(nm)				Fc(%)	Fa(%)	Xc(%)	Single fiber diameter(um)
	020	021	110	111	020	021	110	111				
1.4	8.380	6.228	9.739	7.490	2.573	2.307	2.231	1.760	85.46	78.76	60.26	13.30
1.6	7.803	6.208	8.446	7.196	2.569	2.308	2.231	1.757	87.66	80.19	61.43	13.06
1.8	7.750	6.194	8.458	6.800	2.567	2.306	2.228	1.755	89.29	82.57	62.88	12.69
2.0	7.676	5.816	8.403	6.644	2.568	2.302	2.228	1.753	92.86	86.83	66.34	11.83
2.1	7.378	5.787	8.690	6.607	2.590	2.318	2.248	1.765	95.22	90.99	67.07	11.40

Based on the above conclusions, it can be concluded that the optimal drawing temperature for PBS fibers was 40°C and the optimal heat-setting temperature was 80°C. An increase in the draw ratio results in a reduction of the average crystallite size of the fibers, promoting their orientation and crystallization.

Table S7. Mechanical properties and crystallinity of PBS-POY at different spinning temperatures.

ST(℃)	Linear density（dtex）	Breaking strength（cN/dtex）	SD	Initial modulus（cN/dtex）	SD	Elongation at break（%）	SD	Melting enthalpy（J/g）	Xc (%)
195	60.8	2.09	0.089	13.99	0.085	93.8	5.069	66.83	60.48
205	61.0	2.04	0.104	13.70	0.088	98.0	6.640	64.42	58.30
215	59.1	1.76	0.038	12.30	0.108	107.9	7.087	61.51	55.67

Table S8. The microstructure of PBS-POY at different spinning temperatures.

Spinning temperatures(℃)	Long period(nm)	Thickness of amorphous region (nm)	Thickness of crystalline region (nm)
195	7.80	3.12	4.68
205	7.93	3.10	4.83
215	8.05	3.10	4.95

Table S9. Mechanical properties and crystallinity of PBS-POY at different spinning speeds.

SS (m/min)	Linear density（dtex）	Breaking strength（cN/dtex）	SD	Initial modulus（cN/dtex）	SD	Elongation at break（%）	SD	Melting enthalpy（J/g）	Xc (%)
1200	129.2	0.74	0.059	9.68	0.178	90.5	5.232	61.11	55.30
1500	105.5	1.04	0.028	10.05	0.095	181.9	9.517	61.82	55.95
2000	73.2	1.60	0.086	12.57	0.103	124.2	4.357	63.47	57.44
2300	66.4	1.68	0.086	13.25	0.092	103.2	1.864	65.22	59.02
2500	60.8	2.09	0.089	13.99	0.085	93.8	5.069	66.83	60.48

Table S10. The microstructure of PBS-POY at different spinning speeds.

Spinning speeds(m/min)	Long period(nm)	Thickness of amorphous region (nm)	Thickness of crystalline region (nm)
1200	8.60	3.37	5.23
1500	8.18	3.26	4.92
2000	7.98	3.14	4.84
2300	7.88	3.18	4.70
2500	7.80	3.12	4.68

Table S11. Mechanical properties and crystallinity of PBS-POY at maximum draw ratio for different spinning speeds.

Spinning speeds	Draw ratio	Linear density（dtex）	Breaking strength（cN/dtex）	SD	Elongation at break（%）	SD	Melting enthalpy（J/g）	Xc (%)
1200	1.5	91.4	0.98	0.023	18.2	0.766	62.73	56.77
1500	2.1	51.7	2.52	0.090	17.1	0.516	77.11	69.78
2000	1.8	50.4	2.55	0.115	16.9	0.933	77.52	70.15
2300	1.7	43.0	2.66	0.122	17.1	1.756	77.81	70.42
2500	1.6	41.6	2.72	0.149	16.0	0.693	78.52	71.06

Table S12. The microstructure of PBS-POY at maximum draw ratio for different spinning speeds.

Spinning speeds' draw ratio	Long period(nm)	Thickness of amorphous region (nm)	Thickness of crystalline region (nm)
1200-1.5	8.55	3.36	5.19
1500-2.1	9.07	3.53	5.54
2000-1.8	9.22	3.63	5.59
2300-1.7	9.28	3.70	5.58
2500-1.6	9.99	3.88	6.11

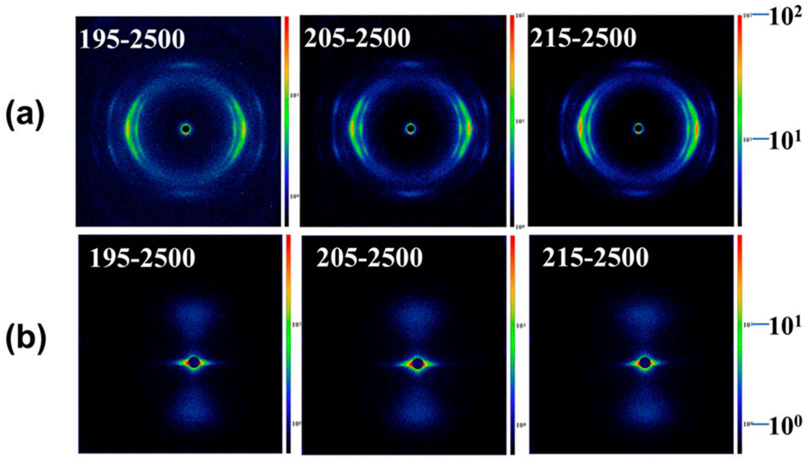

Figure S5. 2D-WAXS and SAXS images of PBS-POY at different spinning temperatures. (**a**)WAXS and (**b**)SAXS.

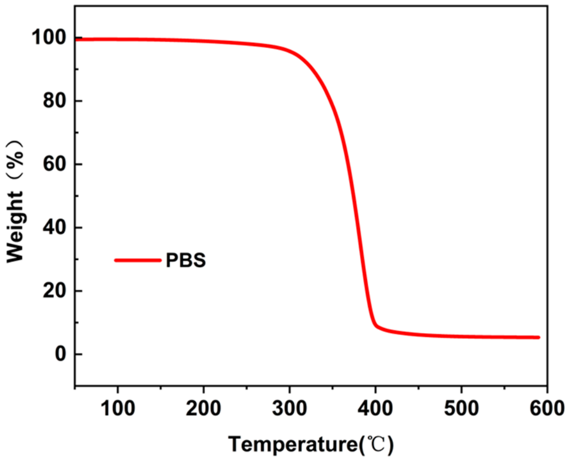

Figure S6. TGA curve of PBS spinning raw material.

Table S13. Thermal stability parameters of PBS spinning raw material.

**Sample**	**Temperatures（℃）of weight loss**		
	5%	10%	50%
**PBS**	309.88	330.93	378.76
